# Signal transducer and activator of transcription 3 is involved in cell growth and survival of human rhabdomyosarcoma and osteosarcoma cells

**DOI:** 10.1186/1471-2407-7-111

**Published:** 2007-06-28

**Authors:** Chun-Liang Chen, Abbey Loy, Ling Cen, Christina Chan, Fu-Chuan Hsieh, Gong Cheng, Bryant Wu, Stephen J Qualman, Keita Kunisada, Keiko Yamauchi-Takihara, Jiayuh Lin

**Affiliations:** 1 Center for Childhood Cancer, Columbus Children's Research Institute and Department of Pediatrics, The Ohio State University, Columbus, OH 43205, USA; 2 Biochmietry Program, The Ohio State University, Columbus, OH 43205, USA; 3 Integrated Biomedical Science Graduate Program, The Ohio State University, Columbus, OH 43205, USA; 4 Department of Molecular Medicine, Osaka University Graduate School of Medicine, Osaka University, 2-2 Yamadaoka, Suita, Osaka 565-0871, Japan

## Abstract

**Background:**

*Stat3 *has been classified as a proto-oncogene and constitutive Stat3 signaling appears to be involved in oncogenesis of human cancers. However, whether constitutive Stat3 signaling plays a role in the survival and growth of osteosarcomas, rhabdomyosarcomas, and soft-tissue sarcomas is still unclear.

**Methods:**

To examine whether Stat3 is activated in osteosarcomas, rhabdomyosarcomas and other soft-tissue sarcomas we analyzed sarcoma tissue microarray slides and sarcoma cell lines using immunohistochemistry and Western blot analysis, respectively, with a phospho-specific Stat3 antibody. To examine whether the activated Stat3 pathway is important for sarcoma cell growth and survival, adenovirus-mediated expression of a dominant-negative Stat3 (Y705F) and a small molecule inhibitor (termed STA-21) were used to inhibit constitutive Stat3 signaling in human sarcoma cell lines expressing elevated levels of Stat3 phosphorylation. Cell viability was determined by MTT assays and induction of apoptosis was analyzed by western blotting using antibodies that specifically recognize cleaved caspases-3, 8, and 9.

**Results:**

Stat3 phosphorylation is elevated in 19% (21/113) of osteosarcoma, 27% (17/64) of rhabdomyosarcoma, and 15% (22/151) of other soft-tissue sarcoma tissues as well as in sarcoma cell lines. Expression of the dominant-negative Stat3 and treatment of STA-21 inhibited cell viability and growth and induced apoptosis through caspases 3, 8 and 9 pathways in human sarcoma cell lines expressing elevated levels of phosphorylated Stat3.

**Conclusion:**

This study demonstrates that Stat3 phosphorylation is elevated in human rhabdomyosarcoma, osteosarcomas and soft-tissue sarcomas. Furthermore, the activated Stat3 pathway is important for cell growth and survival of human sarcoma cells.

## Background

The signal transducer and activator of transcription (STAT) protein family is a group of related proteins that play a role in relaying signals from cytokines and growth factors [1,2]. Many cancers are strongly associated with constant activation of STATs, in particular Stat3 [3,4]. In normal tissues, Stat3 is widely expressed but its transient activation is tightly regulated by SH2-containing tyrosine phosphotases (SHP1 and SHP2), protein inhibitors of activated STATs (PIAS), and suppressors of cytokine signaling proteins/extracellular signaling regulated kinase (SOCS/ERK) cascades as revealed in the Janus associated kinase (JAK)/STAT paradigm [5-7]. In a variety of human cancers, the imbalance among these signaling pathways leads to constitutive activation of Stat3 that is sufficient to induce cell tumorgenesis [8]. Stat3 is also involved in the initiation and promotion of cancers and angiogenesis [9,10]. Targeting the constitutive Stat3 pathway has shown promise in inducing cancer cell death and restricting tumor growth [11-13]. Persistently, activation of Stat3 has become an attractive cancer therapy target [1,4].

Rhabdomyosarcomas, osteosarcomas, and other soft-tissue sarcomas are reported as childhood and adult cancers and their causes remain largely unknown. Rhabdomyosarcoma is the most common soft tissue sarcoma of childhood. Based on histological criteria, it can be classified into two major subtypes, alveolar rhabdomyosarcoma (ARMS) and embryonal rhabdomyosarcoma (ERMS). Although Stat3 is known to be activated in other cancer types, Stat3 activation in osteosarcomas, rhabdomyosarcomas, and soft-tissue sarcomas is still unclear. Further, it is also not clear what role of Stat3 may play in cell growth and survival in human sarcoma cells, including osteosarcoma and rhabdomyosarcoma cells.

Here we present evidence that activated Stat3 is detected in osteosarcoma, rhabdomyosarcoma, and soft-tissue sarcoma tissues and cell lines. Thereafter, we hypothesized that inhibition of Stat3 should lead to suppression of osteosarcoma and rhabdomyosarcoma cell growth. We targeted the activated Stat3 signaling pathway using a dominant negative Stat3 Y705F (dnStat3) and STA-21, a small molecule inhibitor [13,14]. Inhibition of the Stat3 pathway suppressed cell growth of osteosarcoma and rhabdomyosarcoma cell lines *in vitro*. Moreover, blocking of constitutively active Stat3 pathway induced apoptosis through caspases 3, 8 and 9. Taken together, Stat3 may serve as a therapeutic target in human osteosarcomas and rhabdomyosarcomas.

## Methods

### Cell lines

Osteosarcoma (Saos-2, U2OS, and SJSA), rhabdomyosarcoma (RH30, RH3 and RD2), leiomyosarcoma (SK-LMS-1), human foreskin fibroblast (HFF), and human skeletal muscle myoblast (HSMM) cell lines were purchased from American Type Culture Collection (ATCC). CW9019, a rhabdomyosarcoma cell line, was a gift from Dr. Fred Barr (Department of Pathology, University of Pennsylvania). All cell lines were maintained in 1× DMEM supplemented with 10% fetal bovine serum (FBS), 100 U/ml penicilin/streptomycin/amphotericin B (Fisher Scientific International) at 37°C, aired with 5% CO_2_. HSMM cells were grown in SkBM-2 basal medium supplemented with SkGM-2 singleQuots kit according to the manufacture's protocol (Cambrex Bio Science, Walkersville, MD, USA).

### Cancer tissue microarray immunohistochemistry

To examine whether Stat3 is activated in rhabdomyosarcoma, osteosarcomas, and other soft-tissue sarcomas, we stained osteosarcoma (n = 113), rhabdomyosarcoma (n = 64) and other soft-tissue sarcoma (n = 87) tissue samples on tissue microarray slides from different providers (Biopathology Center of Columbus Children Research Institute, Biomax, and Cybrdi) using immunohistochemistry with a p-Stat3(Y705)-specific monoclonal antibody (Cell Singling Tech., Danvers, MA). The immunohistochemistry method was described previously [5,15]. Nuclear p-Stat3 expression levels were scored as 0, 1, 2, and 3 according to the immunohistochemical staining intensity. The nuclear staining intensity was scored on the following scale: 0, no staining; 1, weak staining; 2, moderate staining; and 3, intense staining. Since all the normal tissues stained were scored as 0 and occasionally 1, samples stained with scores 0 or 1 were considered as negative, whereas sarcoma samples with scores 2 and 3 were graded as positive. The intensity of immunostaining was evaluated only when more than 50% nuclei showed p-Stat3 expression. Scoring of immunostaining intensity was completed by two to three independent observers (F. H., G. C., and J. L.). Discrepant scores between the two or three observers were rescored to arrive at a single final score. Light microscopic images were documented using a LEICA DM-4000B fluorescent microscope (Leica Microsystems, Bannockburn, IL) with an attached Diagnostic RT-KE 2 MP digital camera (Diagnostic Instruments, Sterling Heights, MI).

### Western blot

Cells were collected at 4°C in cold harvest buffer supplemented with proteinase inhibitor cocktails and spun down at 3000 × g for 5 min. Cell pellets were lysed in RIPA lysis buffer as described previously [15]. Protein concentrations were quantitated using BCA protein assay kit from Pierce, Inc. (Rockford, IL) according to the manufacture's protocol. Fifty or 100 μg of cellular proteins were resolved on 10% PAGE gels in electrophoresis buffer and transferred to Hybond™-p membrane (Amersham Biosciences, Piscataway, NJ) using transfer buffer with a constant 100 V. The membranes were then blocked using 5% nonfat dry milk in TBST (Tris-HCl, pH7.5, Tween, 0.1%) for 30 min at room temperature (RT) and were incubated with primary antibody over night at 4°C or for 1 hour at RT using concentrations recommended by the manufacture. The membranes were washed three times in 1× TBST for 5 min each time. Proteins of interest were visualized using an ECF™ western blotting kit (Amersham Biosciences, Piscataway, NJ) according to the manufacture's protocol. Incubation of secondary antibody and anti-fluorescein were carried out both in presence of 1× TBST with 2% nonfat dry milk. The fluorescent signals were scanned and documented using a Storm 860 scanner (Molecular Dynamics, Sunnyvale, CA). Antibodies were purchased separately and used for Western blots of FLAG (Sigma, St. Louis, MO, USA), GAPDH (Chemicon International, Temecula, CA), Stat3, and p-Stat3 (Y705) (Cell Signaling Tech., Danvers, MA).

### Transduction of dominant negative Stat3 Y705F in cancer cells

The construction and infection of recombinant Adenovirus/CMV-dnStat3 Y705F (rAd/dnStat3) is described previously [14,16]. DnStat3 was generated from Stat3 by changing the tyrosine at position 705 into phenylalanine. Its protein product cannot be activated through tyrosine phosphorylation that is crucial for dimerization. The clone is tagged with a FLAG marker. About 2 × 10^5 ^U2OS, SaoS2, SJSA, RD2 and RH30 cells were transduced with rAd/dnStat3 or a negative control viral vector, rAd/CMV-eGFP (rAd/eGFP) (Applied Viromics, Fremont, CA) with multiplicities of infection (moi) of 400, 100, and 10 based on TCID50 using 293T cells. For cell growth experiments, cells in 5 random fields of view (100× magnification) were enumerated on days 2, 4, and 6 post-infection of rAd/eGFP and rAd/dnStat3. Cell growth rates were presented as percentages of untransduced controls. Each data point was averaged from triplicate experiments.

### Treatment of STA-21 and cell viability assay

Approximately 5000 RD2 and RH30 cells were grown in 100 μl 10% FBS-supplemented DMEM medium in 96-well flat-bottomed plates overnight. Treated cells were exposed to STA-21 (30 μM) that was dissolved in dimethyl sulfoxide (DMSO) before being added to the medium. Cell viability was analyzed by the MTT [3-(4, 5-dimethyl-2-thiazolyl)-2, 5-diphenyl-2H-tetrazolium bromide] (Sigma, St. Louis, MO, USA) assay in three replicates. At the endpoint, cells were treated with MTT (1 mg/ml) for 3–4 hours. Colorimetric quantification was determined by an EL808 Ultra Micro-plate Reader (Bio-Tek Instruments, Inc) after the addition of formazan dissolved in 25% N, N-dimethylformamide and 10% SDS under light-proof conditions overnight.

### Caspases 3, 8, and 9 immuno-fluorescent staining and acridine orange staining

1 × 10^5 ^cells (U2OS or RD2) were seeded on sterile coverslips in a 6-well plate overnight. The cells were transduced by either rAd/eGFP or rAd/dnStat3 for 3 or 4 days and then fixed using methanol/acetone (v:v = 1:1). Three washes followed the fixation using 1× PBS buffer. During the third wash, the coverslips were transferred to a new 6-well plate. For immuno-fluorescent staining, the cells were blocked in 1× PBS with 10% normal horse serum for 1 hour and incubated with primary rabbit antibodies that recognize cleaved-caspase-3 (Asp175), cleaved-caspase-8 (Asp374), or cleaved-caspase-9 (Asp330) (Cell Singling Tech., Danvers, MA) with 1:100, 1:50, and 1:100 dilutions, respectively. Excess antibodies were removed using 3 washes of 1× PBS with constant agitation, 10 minutes for each wash. Secondary goat anti-rabbit IgG(H+L) Alexa Fluo^R ^594 antibody (Invitrogen, Carlsbad, CA) (1:1000 dilution) was incubated with 1% bovine serum albumin (BSA) in 1× PBS for 1 hour at RT. Unbound antibody was washed off three times using 1× PBS. Nuclei were counter-stained using 4'-6-Diamidino-2-phenylindole (DAPI) (100 ng/ml) in distilled H_2_O for 5 min and then rinsed three times with 1× PBS, 10 min for each wash. The cleaved caspase positive cells were scored from three independent fields of view (100× magnification) and presented in averaged percentages of total cells (DAPI staining) with standard deviations from triplicate experiments.

Acridine orange staining was previously described[17]. The cells were incubated with 1 mg/ml acridine orange (Sigma, St. Louis, MO) for 15 min before 3 washes of 1× PBS. The fluorescence and phase-contrast microscopic photographs were documented using LEICA DM-IRB inverted fluorescent microscope (Leica Microsystems, Bannockburn, IL) with an attached Diagnostic RT-SE6 monochrome digital camera (Diagnostic Instruments, Inc, Sterling Heights, MI).

## Results

We demonstrated that the levels of Stat3 phosphorylation is elevated in human osteosarcomas, rhabdomyosarcomas and other soft-tissue sarcomas tissues. Stat3 signaling pathway plays a role in the cell growth and survival of human sarcomas cells because our data also showed that blocking constitutive Stat3 signaling in sarcoma cells induces apoptosis and growth inhibition. Inhibition of Stat3 signaling in sarcomas may represent an effective new treatment strategy for this type of human cancer.

### Elevated Stat3 phosphorylation in rhabdomyosarcoma, osteosarcoma and other soft-tissue sarcoma tissues and cell lines

**Figure 1:**
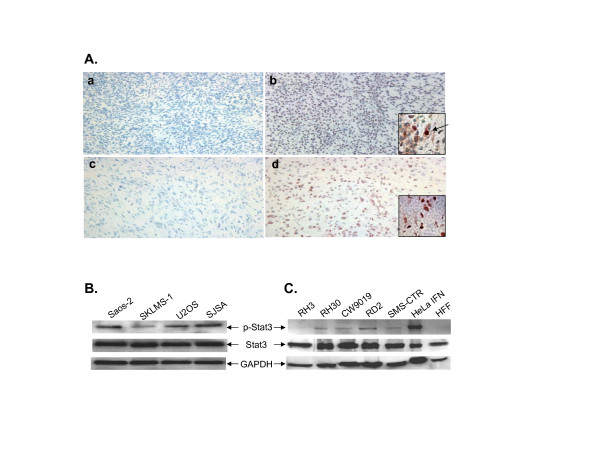
Stat3 phosphorylation is elevated in rhabdomyosarcoma, osteosarcoma and other soft-tissue sarcoma tissues and cell lines. (A) Immunohistochemical staining in sarcoma tissues: a. normal skeletal muscle tissue, b. alveolar rhabdomyosarcoma, c. normal osteo tissue, d. osteosarcoma. The nuclei were counterstained with hematoxylin blue. Image magnifications are 100×. Images of higher magnification (400×) are shown in the insets of b and c. The arrow indicates a cell with nuclear and cytoplasmic p-Stat3 staining. Western blots also show elevated p-Stat3 in (B) osteosarcoma and leiomyosarcoma cell lines and (C) rhabdomyosarcoma cell lines.

**Figure 2:**
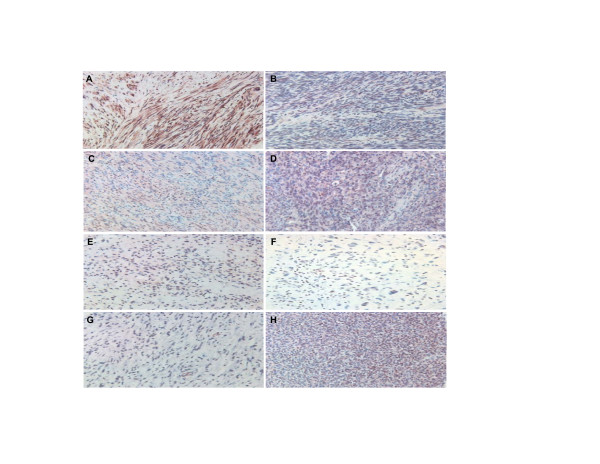
Anti-p-Stat3 immunohistochemistry shows that Stat3 phosphorylation is elevated in other soft-tissue sarcomas. (A) neurofibroma, (B) synovial sarcoma, (C) neurilemmoma, (D) angio sarcoma, (E) myxoid liposarcoma, (F) malignant fibrous histiocytoma, (G) myxoid malignant fibrous histocytoma, (H) hemagiopericytoma. The nuclei were counterstained with hematoxylin blue. All image magnifications are 100×.

Our results indicated that Stat3 phosphorylation levels were elevated in osteosarcoma, rhabdomyosarcoma and other soft-tissue sarcoma tissues and cell lines (Figure 1 &2). The sarcoma tissues used here were classified according to clinicopathological data in Table 1 and 2. Osteosarcoma tissue microarray slides had a total of 113 specimens. Soft-tissue sarcoma microarray slides had a total of 151 specimens (Table 1). Rhabdomyosarcoma is the most common soft tissue sarcoma of childhood. Based on histological criteria, it can be classified into two major subtypes, alveolar rhabdomyosarcoma (ARMS) and embryonal rhabdomyosarcoma (ERMS). Rhabdomyosarcoma tissue microarray slides had a total of 64 specimens in which 32 of them were ARMS and another 32 specimens were ERMS. The patient ages of these cases were between 0 and 19 (Table 2). Normal tissues did not stain for p-Stat3 (Figure 1A: a &1c) and sarcoma tissues stained positively in nuclei, cytoplasm, or both (Figure 1A: b, d and insets in b, d). The percentages of p-Stat3 positive samples were 19% (21/113) of osteosarcoma, 27% (17/64) of rhabdomyosarcoma, and 15% (22/151) of other soft-tissue sarcoma samples.

**Table 1:** Clinicopathological parameters of Osteosarcoma and soft-tissue sarcoma analyzed

Clinicopathological parameters		Numbers (%)
Gender (total 264)		
	Female	113 (42.7)
	Male	151 (57.2)
		
Age (years) (total 264)	1–20	65 (24.6)
	21–40	98 (36.9)
	41–60	55 (20.7)
	61–80	42 (15.8)
	81–100	4 (1.5)
		
Grade (total 15)	I	14 (93.3)^a^
	III	1 (6.7)^a^
		
Histology (total 264)	Osteosarcoma	113 (42.5)
	Liposarcoma	32 (12.1)
	Chondrosarcoma	28 (10.6)
	Histiosarcoma	26 (9.8)
	Leiomyosarcoma	19 (7.1)
	Fibrosarcoma	16 (6.0)
	Neurilemmoma	12 (4.4)
	Neurofibroma	4 (1.5)
	Synovial Sarcoma	4 (1.5)
	Hemangiosarcoma	3 (1.1)
	Alveolar Sarcoma	2 (0.75)
	Adenocarcinoma	1 (0.38)
	Epithelioid hemangioendothelioma	1 (0.38)
	Malignant Glomus Thigh Tumor	1 (0.38)
	Malignant Schwannoma	1 (0.38)
	Malignant Tienosynovial Tumor	1 (0.38)
	Sacrococcygeal Melanoma	1 (0.38)

^a ^Grade I: 14 chondrosarcomas in cartilages; Grade III: one adenocarcinoma in fibrous tissue. Grade information is not available in other sarcoma specimens

**Table 2:** Clinicopathological parameters of Alveolar (ARMS) and Embryonic (ERMS) patients used

Variable	Number of patients (32 each type)	
	ARMS	ERMS
Gender		
Male	13	24
Female	19	8
Age (years)		
0–4	11	18
5–14	14	10
15–19	7	4
Mean	9.1	5.8
Median	10	4
Stage		
I	5	7
II	5	4
III	15	14
IV	7	7
Primary site		
Head and Neck	11	10
Extremity	12	3
Genitourinary	4	9
Others	5	10

We also investigated the status of p-Stat3 in sarcoma cell lines. Analysis of Stat3 phosphorylation in these cells lines was carried out using Western blots with GAPDH as an internal protein loading control (Figure 1B &1C). Western blots with a p-Stat3 specific antibody revealed that Stat3 was phosphorylated in several rhabdomyosarcoma, osteosarcoma, and leiomyosarcoma cell lines. These included RD2, RH30, CW9019, SMS-CTR, Saos-2, SKLMS-1, U2OS, SJSA, as well as IFN-γ-treated HeLa cells serving as a positive control. P-Stat3 levels in RH3 and a negative control cell line, HFF, were relatively low or undetectable. However, these two p-Stat3 negative cell lines contained similar levels of total Stat3 with the other p-Stat3 positive cell lines. Elevated Stat3 phosphorylation crucial for Stat3 activation was observed in most of the sarcoma cell lines we screened.

### rAd-mediated transduction of dnStat3 in rhabdomyosarcoma and osteosarcoma cell lines

**Figure 3:**
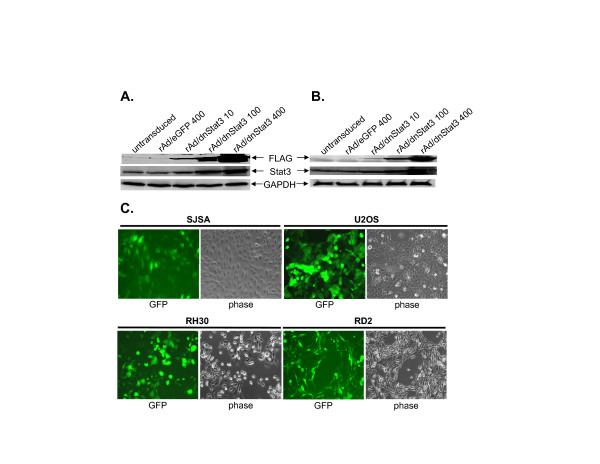
Western blots show dnStat3 expression in a dose-dependent manner in (A) SJSA osteosarcoma and (B) RD2 rhabdomyosarcoma cell lines. (C) Nearly 100% transduction efficiency of rAd/GFP (moi of 400) on SJSA, U2OS, RH30 and RD2 cell lines at day 4 post-infection. GFP: green fluorescent protein expression images. Phase: phase-contrasted images. All image magnifications are 100×.

Since elevated levels of Stat3 phosphorylation was found in sarcoma tissues and cell lines, we subsequently investigated the role activated Stat3 may play in cell growth or survival of sarcoma cell lines. We introduced dnStat3 into rhabdomyosarcoma and osteosarcoma cell lines using an adenoviral vector delivery system. RD2 and SJSA cells were infected with rAd/dnStat3 (moi = 10, 100, and 400). FLAG-tagged dnStat3 expression levels in sarcoma cells were detected in Western blots probed with an anti-FLAG antibody. Two days post-infection, dnStat3 was expressed in SJSA and RD2 cells in a dose-dependent manner, but not in untransduced cells and cells transduced with rAd/eGFP (moi = 400) (Figure 3A &3B). The transduction efficiency of rAd vector on these cells was determined by infection of rAd/eGFP. Greater than 90% of cancer cells showed green fluorescence by day 4 post-infection with rAd/eGFP (moi = 400) (Figure 3C and Saos-2 data not shown).

### Targeting Stat3 signaling pathway using dnStat3 and STA-21 suppressed cell growth and viability in rhabdomyosarcoma and osteosarcoma cells

Osteosarcoma and rhabdomyosarcoma cell growth and viability were significantly suppressed in the presence of dnStat3 or STA-21. Sarcoma cells were transduced with either rAd/eGFP or rAd/dnStat3 (moi = 400). Growth of cells with/without transduction was normalized to untransduced controls at days 2, 4 and 6 post-transduction (Figure 4A). The growth rates of untransduced cells were set at 100%. There were minor adverse effects by rAd/eGFP on cell growth as observed in osteosarcoma and rhabdomyosarcoma cells when moi of 400 was used. However, all sarcoma cells transduced with rAd/dnStat3 showed dramatic reduction in cell growth being less than 20 or 40% of untransduced controls (day 4 or 6 post-infection) (Figure 4A). To investigate whether the dnStat3 cell growth inhibition effects were specific to sarcoma cells, a normal human skeletal muscle myoblast cell line (HSMM) was transduced with the same dose of rAd/dnStat3 and rAd/eGFP (moi = 400). Interestingly, at day 6 post infection, there were no observable changes in cell growth and morphology of HSMM (data not shown).

STA-21 is a novel small inhibitor that prevents Stat3 from dimerization, translocation into the nucleus, and its signaling pathway [13]. The cell viability of RH30 and RD2 were also greatly reduced after 4 and 5 days' exposure to STA-21 (Figure 4B). Approximately 12% and 48% untreated cell viability were detected in RH30 and RD2 cells, respectively, exposed to 30 μM STA-21 for 4 days. After 5-day treatment, STA-21 inhibitory effects were even more significant that viabilities of drug-treated RH30 and RD2 cells were only 1.8% and 11% of the untreated, respectively.

### Apoptosis induced by dnStat3 and STA-21 through caspase cleavage pathways

Rhabdomyosarcoma and osteosarcoma cell lines transduced with rAd/dnStat3 not only suffered cell growth and viability inhibition but also total cell number reduction suggesting that cell death was induced by the expression of dnStat3 (Figure 4C and data not shown). In addition, massive accumulation of vacuoles occurred in the cytoplasm of these cells (Data not shown). These prompted us to explore if necrosis or apoptosis contribute to the cell death. In order to investigate the mechanism underlying the rhabdomyosarcoma and osteosarcoma cell death, RD2 and U2OS cells were fixed at day 4 post transduction of rAd/eGFP or rAd/dnStat3. The fixed cells were then subjected to acridine orange staining and anti-cleaved-caspase immuno-fluorescent staining for necrosis and apoptosis evaluations, respectively. For acridine orange staining, there was no difference between control cells and transduced cells with rAd/dnStat3 (data not shown) indicating that necrosis is not involved. For apoptosis evaluation, there was no observable difference in cleaved caspase immuno-staining among negative controls (untransduced or transduced with rAd/eGFP) in U2OS and RD2 cells lines (Figure 5A &5B). However, cell death caused by the transduction of dnStat3 appeared to be apoptosis as cleaved caspases 3, 8, and 9 were observed in increased portions of dnStat3-expressing RD2 and U2OS sarcoma cells (Figure 5A, B &5C; cleaved caspase 9 staining for U2OS and cleaved caspase 8 staining for RD2, data not shown). In RD2 cells, 4-day dnStat3 expression induced 23%, 44%, and 52% of cells to undergo apoptosis through cleaved caspases 3, 8 and 9, respectively. Similarly in U2OS cells, transduction of dnStat3 caused caspases 3, 8, and 9 cleavages in 27%, 42%, and 63% of cells, respectively (Figure 5C). The apoptotic effect through the blocking of Stat3 signaling in sarcoma cells was further confirmed using STA-21. STA-21-treated rhabdomyosarcoma cells, RH30 and RD2, showed increased portions (58.2% and 35.4%) of cells undergoing apoptosis through the caspase 3 cleavage pathway (Figure 5D &5E). In contrast, normal HSMM cells were not affected by the STA-21 treatment for the same duration (Figure 5D &5E). Apparently, cytotoxicity to normal cells by targeting the Stat3 signaling pathway would be very minimal.

## Discussion

We demonstrated that elevated levels of Stat3 phosphorylation are detected in some sarcoma tissues (15–27%). Phosphorylation at tyrosine 705 is important for the activation of Stat3. The mechanisms underlying the elevated Stat3 phosphorylation in these sarcoma tissues are not clear. There might be constant upstream activation by cytokines and growth factors [1,18], down regulation of counter balancing signal transduction pathways, such as SOCS1, or both [7]. In rhabdomyosarcoma, the fusion protein PAX3-FKHR directly interacts with Stat3 and changes gene expression profiles that are normally regulated by JAK/STAT signaling pathways. This leads to alterations in local cytokine concentrations that inhibit adjacent inflammatory cells and evade immune detection [19]. Activation of Stat3 was reported to be present in Ewing sarcoma family tumors (ESFT) [20]. These previous studies are consistent with our finding that the Stat3 signaling pathway is constitutively activated in rhabdomyosarcomas, osteosarcomas, and other soft-tissue sarcomas.

Our data strongly support that the activated Stat3 pathway could serve as a therapeutic target in rhabdomyosarcoma and osteosarcoma cancers using a dominant negative Stat3 mutant or a small molecule Stat3 inhibitor, STA-21. It has been shown that interference of the Stat3 signaling pathway leads to cancer cell apoptosis and proliferation prohibition [13,21-23]. We targeted activated Stat3 pathways with rAd/dnStat3 and STA-21 in sarcoma cell lines. Suppression of cell growth and cell number reduction were observed in sarcoma cells expressing dnStat3 or exposed to STA-21. Interestingly, no such dnStat3 inhibitory effects were observed in HSMM, normal human skeletal myoblasts. These data support that suppression of cell growth in sarcoma cells is likely due to the antagonizing effects of dnStat3 and STA-21 on the cell proliferation that is promoted by elevated Stat3 phosphorylation.

Transduction of dnStat3 and treatment of STA-21 induces apoptosis in rhabdomyosarcoma and osteosarcoma cells *in vitro*. Compared to untransduced or rAd/GFP-transduced controls, sarcoma cells infected with rAd/dnStat3 showed significant reductions in total cell number and suggested that cell death had occurred. Cell death caused by dnStat3 is apparently caspase-dependent apoptosis, since cells undergoing apoptosis contained activated caspases 3, 8 and 9 as demonstrated by immuno-fluorescent staining. This implies that dnStat3 induces apoptosis through two independent upstream pathways mediated by caspases 8 and 9 that lead to the cleavage of downstream caspase 3. Activation of Stat3 has been shown to enhance cell survival and proliferation of cancer cells and render them resistant to chemotherapeutic drugs and stress through the activation of survival genes and cell-cycle regulated genes [24,25]. We report here a very intriguing phenomenon that dnStat3-induced apoptosis is mediated through both caspase-8 and -9 pathways. Induction mechanisms for apoptosis through caspase 8 and 9 pathways are different [26-30]. The involvement of caspase 8 pathway suggests an autocrine role that dnStat3 transduction may play in apoptosis-bound sarcoma cells. The underlying mechanisms are still elusive and are worth pursuing. The induction of apoptosis by blocking Stat3 pathway in sarcoma cells expressing elevated levels of p-Stat3 is further supported using STA-21 that is an effective Stat3 inhibitor in breast cancer and some other cancer cells [[13] and unpublished data]. Given the low cytotoxicity of Stat3 inhibition to normal cells, targeting Stat3 signaling pathway would be a promising therapeutic strategy for sarcomas in which Stat3 is constitutively activated.

## Conclusion

Stat3 phosphorylation is elevated in human rhabdomyosarcoma, osteosarcomas, and other soft-tissue sarcomas. The Stat3 pathway is involved in cell growth and survival of human rhabdomyosarcoma and osteosarcoma cells. Inhibition of Stat3 signaling in sarcomas may represent an effective treatment strategy for these types of cancer.

## Competing interests

The author(s) declare that they have no competing interests.

## Authors' contributions

CC participated in experiment designs, conducted most of the experiments, contributed to the analysis and interpretation of data, and drafted the manuscript. AL carried out the Adeno-viral dnStat3 experiments. AL, LC, CC, and BW carried out or assisted in the cell viability using MTT assay and Western blotting for phosphorylated Stat3, total Stat3, and cleaved casapses 3, 8, and 9. FH and GC, carried out immunohistochemical staining of tissue microarray slides. SJQ's laboratory designed, constructed and provided rhabdomyosaroma tissue microarray slides as collaboration. KK and K Y-T laboratories designed, constructed and provided rAdv dnStat3 vector as collaboration. All authors read and approved the final manuscript.

## Pre-publication history

The pre-publication history for this paper can be accessed here:



## Supplementary Material

Additional file 1 (A) U2OS, Saos-2, SJSA, RD2 and RH30 cell growth is inhibited by the transduction of dnStat3. (B) Cell viabilities of RH30 and RD2 are also suppressed after 4- and 5-day exposure to STA-21 as shown by a MTT assay. (C) Sarcoma cells are greatly decreased after expressing dnStat3. Y axis is in a log scale. All averages and standard deviations are based on triplicate experiments.

Additional file 2 Expression of dnStat3 in (A) U2OS osteosarcoma and (B) RD2 rhabdomyosarcoma cells induces apoptosis through cleaved caspases 3, 8, and 9 pathways. (C) Cleaved caspase positive cells in U2OS and RD2 are presented in percentages of all cells scored. One of two experiments is shown. (D) Blocking of Stat3 pathway by STA-21 also causes apoptosis in RD2 and RH30 cells but not in HSMM cells through casapase 3 cleavage. (E) Quantification of cleaved caspase 3 positive cells in RH30, RD2 and HSMM cells. Cleaved caspases 3, 8, and 9: anti-cleaved-caspases 3, 8, and 9 immuno-fluorescent staining. DAPI: nuclear staining with DAPI; Phase: phase-contrast images; Unt: untransduced or untreated cells; All image magnifications are 50×.
