# Supplementary figures and images for: Signal transducer and activator of transcription 3 is involved in cell growth and survival of human rhabdomyosarcoma and osteosarcoma cells
Source: BMC Cancer. 2007 Jun 28;7:111. doi: 10.1186/1471-2407-7-111 (PMC1964761; doi:10.1186/1471-2407-7-111)

**A.**

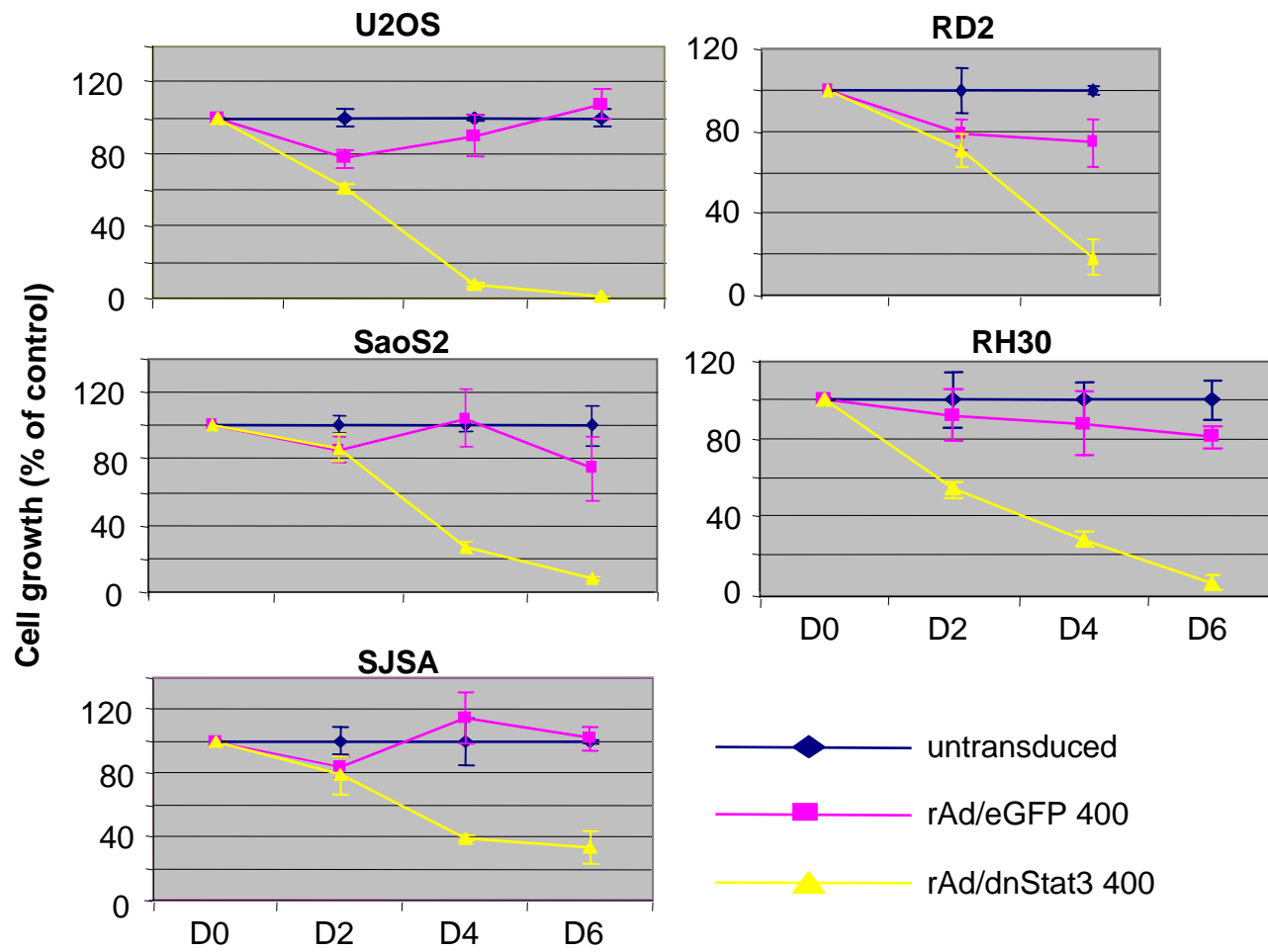

**B.**

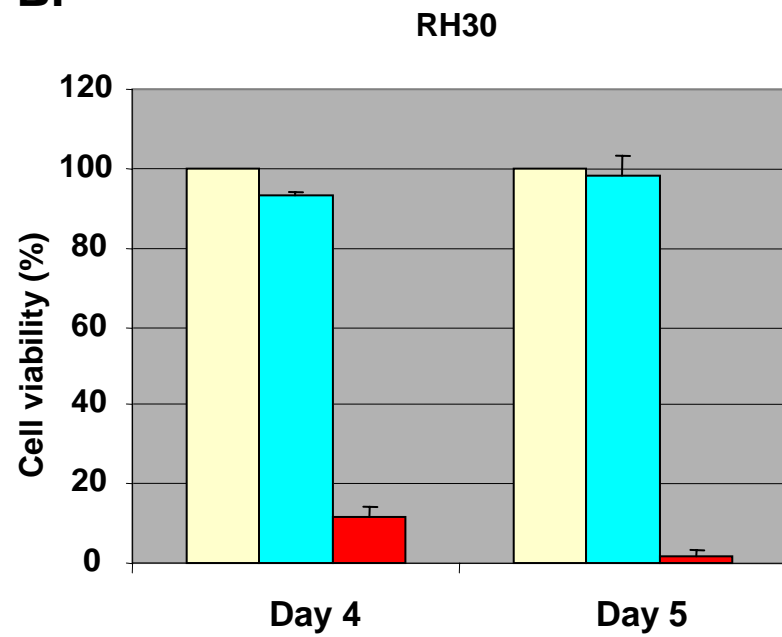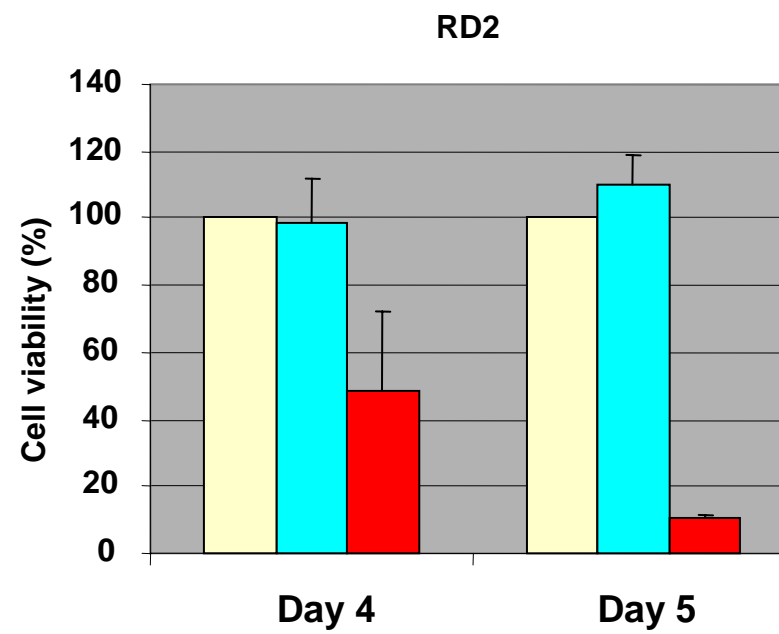

untreated      DMSO      STA-21 30  $\mu$ M

**C.**

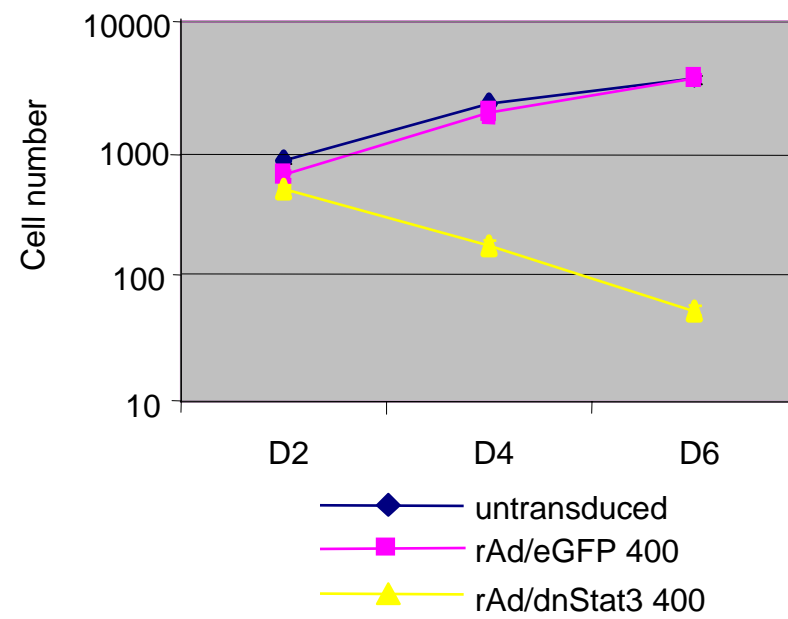

Supplement: Additional file 1 — (A) U2OS, Saos-2, SJSA, RD2 and RH30 cell growth is inhibited by the transduction of dnStat3. (B) Cell viabilities of RH30 and RD2 are also suppressed after 4- and 5-day exposure to STA-21 as shown by a MTT assay. (C) Sarcoma cells are greatly decreased after expressing dnStat3. Y axis is in a log scale. All averages and standard deviations are based on triplicate experiments. [file 1471-2407-7-111-S1.pdf]
