# Supplementary material for: Signal transducer and activator of transcription 3 is involved in cell growth and survival of human rhabdomyosarcoma and osteosarcoma cells
Source: BMC Cancer. 2007 Jun 28;7:111. doi: 10.1186/1471-2407-7-111 (PMC1964761; doi:10.1186/1471-2407-7-111)
Supplement: Additional file 2 — Expression of dnStat3 in (A) U2OS osteosarcoma and (B) RD2 rhabdomyosarcoma cells induces apoptosis through cleaved caspases 3, 8, and 9 pathways. (C) Cleaved caspase positive cells in U2OS and RD2 are presented in percentages of all cells scored. One of two experiments is shown. (D) Blocking of Stat3 pathway by STA-21 also causes apoptosis in RD2 and RH30 cells but not in HSMM cells through casapase 3 cleavage. (E) Quantification of cleaved caspase 3 positive cells in RH30, RD2 and HSMM cells. Cleaved caspases 3, 8, and 9: anti-cleaved-caspases 3, 8, and 9 immuno-fluorescent staining. DAPI: nuclear staining with DAPI; Phase: phase-contrast images; Unt: untransduced or untreated cells; All image magnifications are 50×. [file 1471-2407-7-111-S2.pdf]

A.

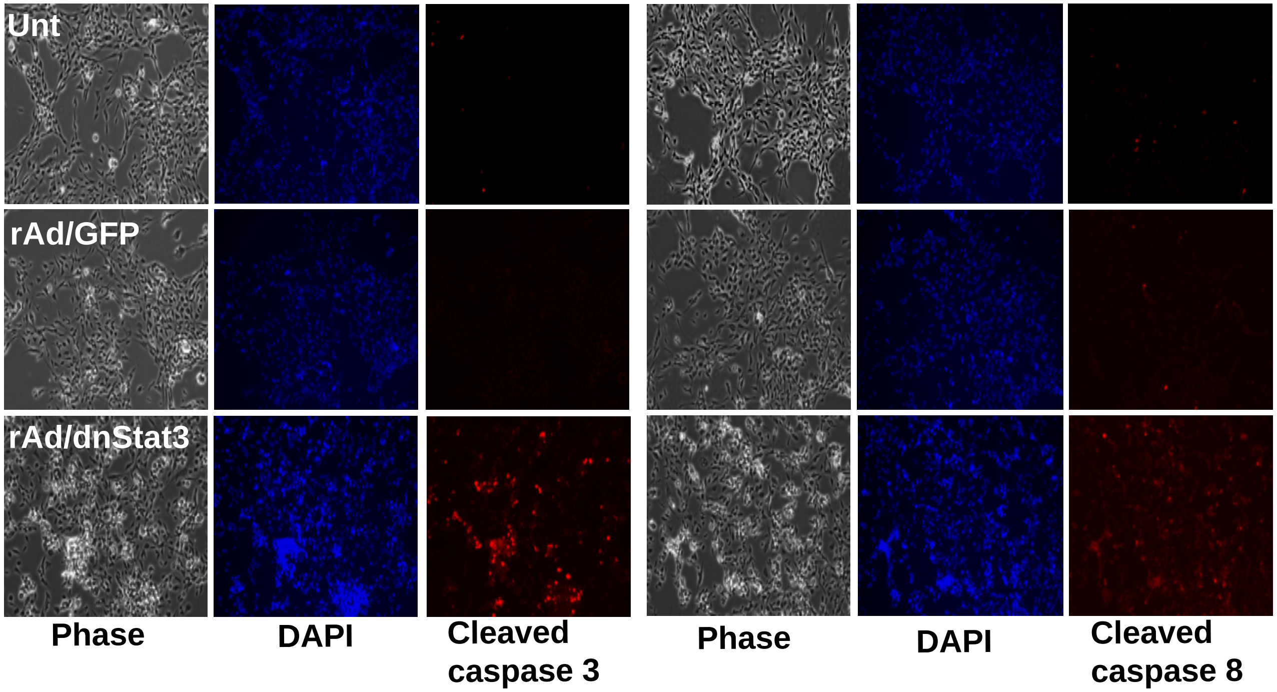

Unt
rAd/GFP
rAd/dnStat3
Phase
DAPI
Cleaved caspase 3
Phase
DAPI
Cleaved caspase 8

B.

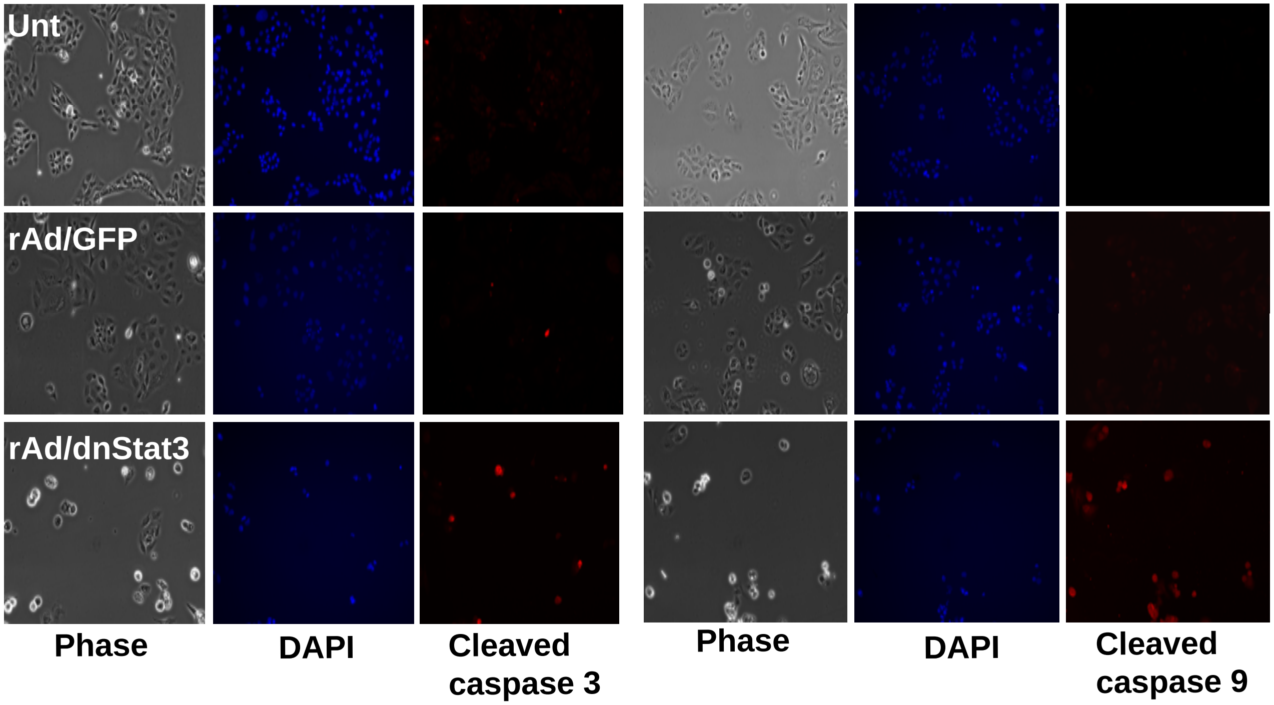
Unt
rAd/GFP
rAd/dnStat3
Phase
DAPI
Cleaved caspase 3
Phase
DAPI
Cleaved caspase 9

C.

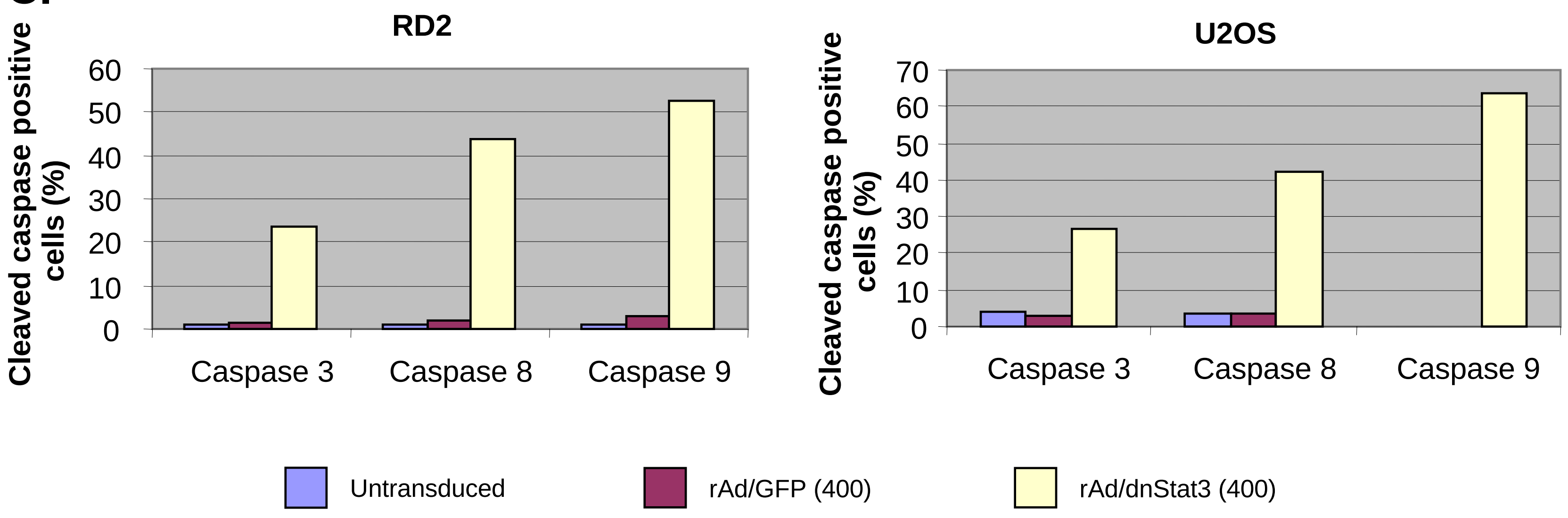
RD2
U2OS
Cleaved caspase positive cells (%)
0
10
20
30
40
50
60
70
Caspase 3
Caspase 8
Caspase 9
Untransduced
rAd/GFP (400)
rAd/dnStat3 (400)

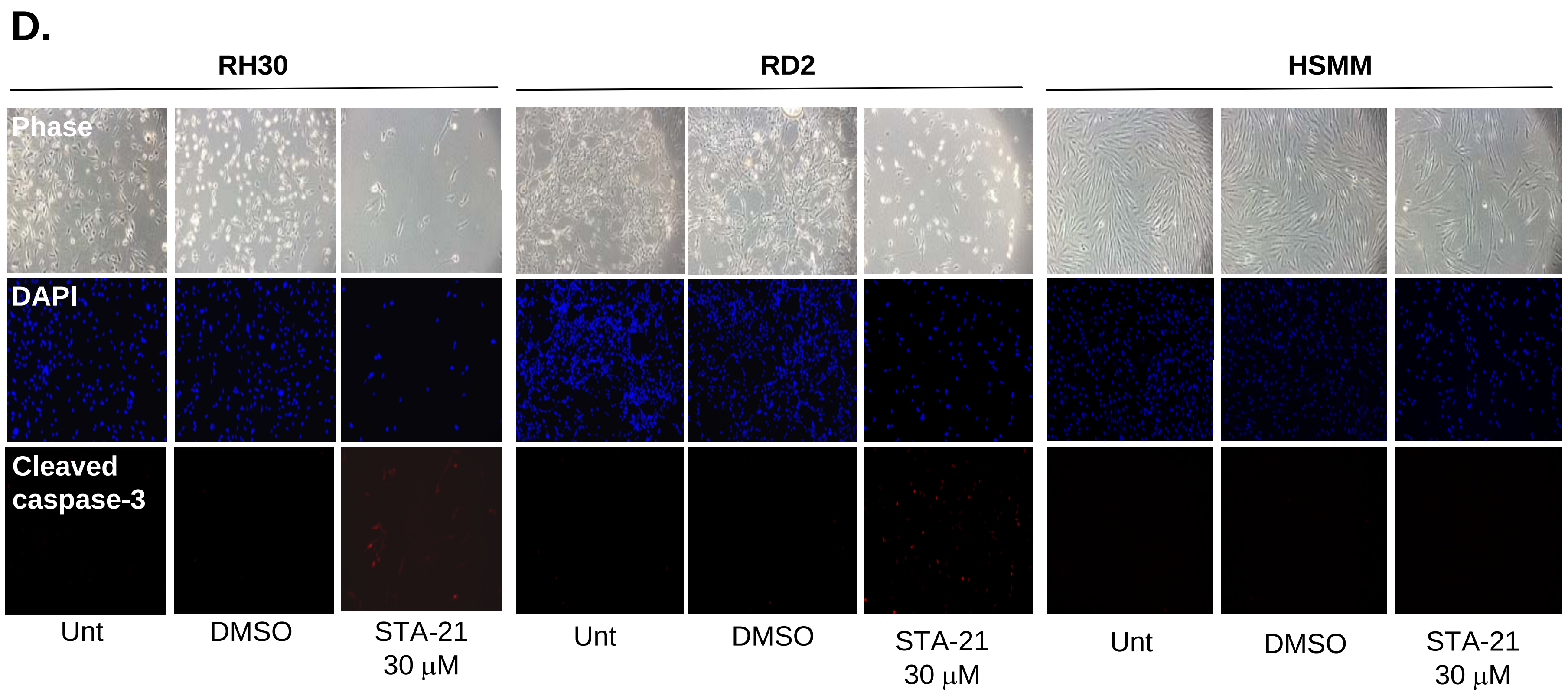
D.
RH30
RD2
HSMM
Phase
DAPI
Cleaved caspase-3
Unt
DMSO
STA-21 30 μM
Unt
DMSO
STA-21 30 μM
Unt
DMSO
STA-21 30 μM

**E.**

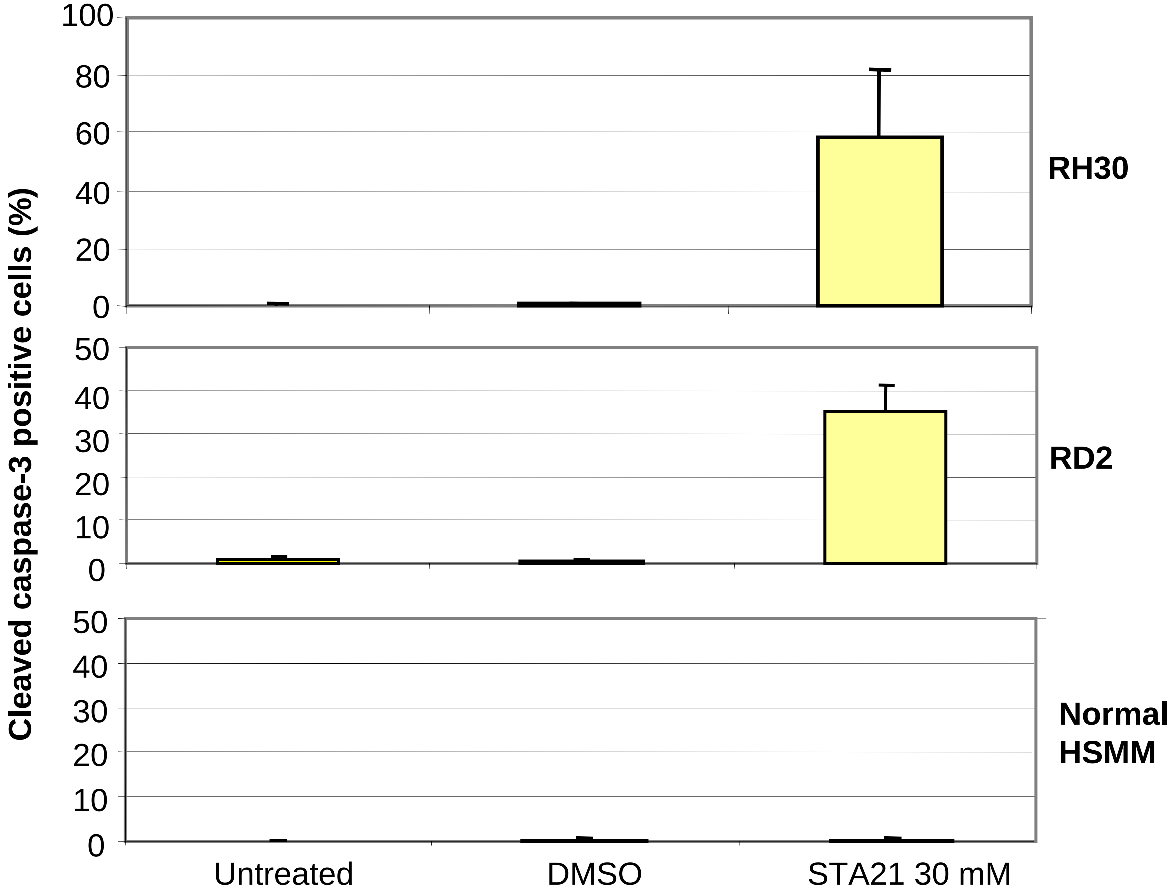
Cleaved caspase-3 positive cells (%)
100
80
60
40
20
0
RH30
50
40
30
20
10
0
RD2
50
40
30
20
10
0
Normal
HSMM
Untreated
DMSO
STA21 30 mM
